# Determining mosquito age using surface-enhanced Raman spectroscopy and artificial neural networks: insights into the influence of origin and sex

**DOI:** 10.1186/s13071-025-06831-x

**Published:** 2025-06-10

**Authors:** Zili Gao, Yuzhen Zhang, Laura C. Harrington, Courtney C. Murdock, Elisabeth Martin, Dalton Manbeck-Mosig, Steve Vetrone, Nicolas Tremblay, Christopher M. Barker, John M. Clark, Lili He, Wei Zhu

**Affiliations:** 1 Department of Food Science, University of Massachusetts, Amherst, MA 01003 USA; 2 Raman, IR, and XRF Core Facility, University of Massachusetts, Amherst, MA 01003 USA; 3 Department of Entomology, Cornell University, Ithaca, NY 14853 USA; 4 School of Veterinary Medicine, University of California Davis, Davis, CA 95616 USA; 5 Greater Los Angeles County Vector Control District, Santa Fe Springs, CA 90670 USA; 6 Department of Veterinary and Animal Sciences, University of Massachusetts, Amherst, MA 01003 USA; 7 Department of Chemistry, University of Massachusetts, Amherst, MA 01003 USA; 8 School of Mathematics, Georgia Institute of Technology, Atlanta, GA 30332 USA

**Keywords:** Mosquito classification, Age prediction, Surface-enhanced Raman spectroscopy, Artificial neural networks

## Abstract

**Background:**

Mosquito-borne diseases, such as malaria, dengue, and Zika, continue to pose significant threats to global health, resulting in millions of cases and thousands of deaths each year. Notably, only older mosquitoes can transmit these diseases. Therefore, accurate age estimation of mosquitoes is vital for targeted interventions and risk assessments. However, traditional methods, such as tracheole morphology analysis, are labor-intensive and have limited scalability. Surface-enhanced Raman spectroscopy (SERS), when coupled with artificial neural networks (ANNs), offers a robust and flexible alternative, facilitating accurate and efficient mosquito age determination even in diverse and complex environmental conditions.

**Methods:**

We analyzed 124 *Aedes aegypti* mosquitoes from California (CA) and Thailand (TH) using SERS, each generating 20 spectra. The ANNs utilized a multilayer perceptron with two hidden layers of 100 neurons and rectified linear unit (ReLU) activation. Classification tasks used cross-entropy loss; regression applied mean squared error. Models were trained with a 70–30 training–validation split and optimized using the Adam optimizer over 10,000 iterations. Performance metrics included accuracy, correlation coefficient (*R*), and root mean square error (RMSE). t-Distributed stochastic neighbor embedding (t-SNE) visualizations and confusion matrices offered additional model insights into effectiveness.

**Results:**

The ANN models demonstrated superior performance in differentiating mosquito age relative to non-ANN methods. For female CA mosquitoes, the models classified ages from day 1 to day 21 with 84% accuracy and predicted age with an *R* of 0.96 and RMSE of 2.18 days. Similarly, the models achieved 86% accuracy and an *R*-value of 0.95 for female TH mosquitoes. While mosquito origin and sex influenced performance, the combined model maintained robust results, achieving 80% accuracy and an *R*-value of 0.93. Implementing a voting mechanism across multiple spectra for each mosquito significantly improved accuracy, increasing classification performance from approximately 80% at the spectrum level to 100% at the mosquito level.

**Conclusions:**

This study demonstrates the effectiveness of SERS combined with ANN for accurate age classification and prediction of *Ae. aegypti* mosquitoes. The models achieved high accuracy across diverse populations, with a voting mechanism enhancing classification to 100%. These findings highlight the potential of SERS-ANN as a reliable tool for vector control and disease surveillance.

**Graphical Abstract:**

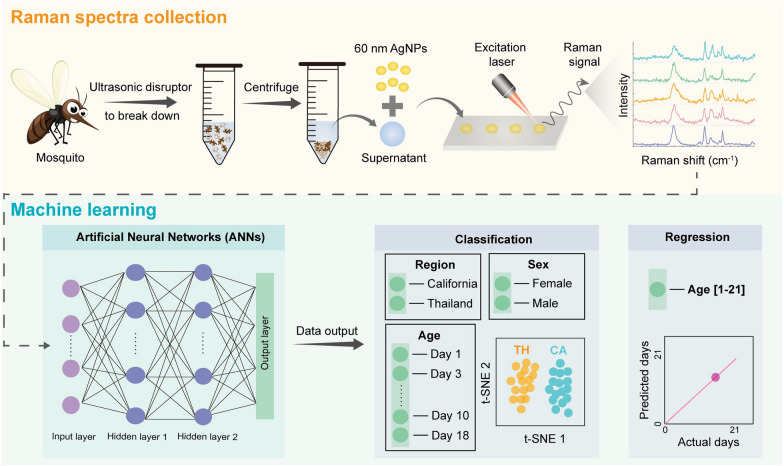

**Supplementary Information:**

The online version contains supplementary material available at 10.1186/s13071-025-06831-x.

## Background

Mosquito-borne diseases are a major global health concern, affecting millions yearly. Malaria impacts hundreds of millions, with an estimated 249 million cases and 608,000 deaths in 2022 [1]. Dengue, another mosquito-transmitted infection, affected over 80 countries in 2023, leading to a historical high of over 6.5 million cases and more than 7300 deaths this year [2]. Other diseases like chikungunya and Zika also contribute to the global health burden [3]. Over half of the world’s population is at risk, highlighting the need for effective disease control to prevent outbreaks. However, vector control faces challenges from insecticide resistance and logistical constraints, necessitating new strategies for risk assessment and targeted interventions [4–6].

Mosquito age plays a critical role in disease transmission, as only older mosquitoes are capable of spreading pathogens [7]. This is due to the extrinsic incubation period—the time required for a pathogen to develop within the mosquito before it can be transmitted to a new host. For instance, in *Aedes aegypti*, the primary vector of dengue virus, this period typically lasts between 8 to 12 days, depending on ambient temperature [8]. Once the virus has completed its incubation, the mosquito becomes infectious and can transmit the disease with each subsequent blood meal. Understanding mosquito age is, therefore, essential for assessing transmission risk, as older mosquitoes are potentially infectious. Additionally, older mosquitoes are more vulnerable to insecticides, highlighting the need for age-specific vector control strategies [9, 10].

Conventional mosquito age estimation methods, such as tracheole morphology and follicular dilatation counting, can indicate previous egg-laying activity but are labor-intensive and imprecise. Hugo et al. [11] found ovarian tracheation and midgut meconium detection reliable for distinguishing nulliparous from parous females, while Polovodova and growth line analysis were inaccurate for multiple age groups. Anagonou et al. [12] validated the Polovodova method using oil injection but found the classical dilaceration method ineffective for multiparous classification. These studies highlight the limitations of morphological techniques, with accuracy varying by method and species.

Gene transcription-based mosquito age grading improves accuracy for population surveillance but faces limitations. Caragata et al. [13] enhanced transcriptional profiling for *Ae. aegypti*, improving accuracy for older mosquitoes but with reduced reliability. Wang et al. [14] identified gene biomarkers for *Anopheles gambiae*, yet environmental and genetic variability affected predictions. Pruszynski et al. [15] estimated *Ae. aegypti* age structure in Florida but emphasized the need for further validation. While promising, transcriptional profiling struggles with accuracy in older mosquitoes, environmental sensitivity, and accessibility relative to morphological or spectroscopic methods.

Surface-enhanced Raman spectroscopy (SERS) is an advanced spectroscopic technique that amplifies Raman signals through interactions with nanoscale metallic surfaces, significantly enhancing sensitivity and enabling the detection of trace biomolecular changes. It provides unique spectroscopic fingerprints for diverse applications, including food safety [16–18], biomedical diagnostics [19–21], and pathogen detection [22–25], making it a powerful tool for molecular analysis and classification. This label-free method provides high specificity and detailed molecular fingerprints, making it a valuable tool for mosquito age grading [26, 27]. Compared with infrared (IR) spectroscopy, which includes near-infrared (NIR) and mid-infrared (MIR) techniques and relies on the absorption of vibrational energy by molecular bonds, SERS offers superior molecular resolution by enhancing Raman scattering signals through interactions with nanoscale metallic surfaces. While IR spectroscopy has demonstrated effectiveness in broadly classifying mosquitoes into very young (< 7 days) or very old (> 7 days) groups [28–32], its limited spectral resolution makes it less suitable for precise age differentiation. In contrast, SERS generates rich spectral data that makes it highly suitable for machine learning (ML) applications [33–36]. By providing detailed molecular fingerprints, SERS enables ML algorithms to identify complex spectral patterns with high precision, enhancing predictive accuracy across various analytical fields. These advantages establish SERS as a powerful tool for data-driven classification and quantitative analysis.

In previous research by our group, Wang et al. [37] developed a method using SERS and chemometrics to accurately determine the age of laboratory-reared *Ae. aegypti* mosquitoes. Building on this, Gao et al. [38] advanced the approach by integrating SERS with artificial neural networks (ANNs) to accurately estimate the age of field-aged *Ae. aegypti* mosquitoes. The SERS-ANN model achieved 86% accuracy in age classification (i.e., categorizing mosquitoes into *discrete* age bins) and demonstrated a root mean square error (RMSE) of 1.9 days and an *R*-value of 0.955 for age prediction (i.e., predicting *continuous* chronological age using a regression model). The integration of ANNs with SERS allows for more accurate, robust, and flexible age grading of mosquitoes, especially when handling complex spectral data from field-aged specimens exposed to varying environmental conditions. This approach marks a significant improvement over traditional linear chemometric methods used with SERS alone.

Building on our previous work, this study aims to evaluate the capability of SERS combined with ANNs for mosquito age analysis while considering the influence of geographic origin (California, USA [CA] and Thailand [TH]) and sex. The origin differences in this study include variations in strain and rearing methods. Using principal component analysis (PCA), partial least squares (PLS), and ANNs, the effectiveness of ML in extracting meaningful patterns from spectral data for age grading was assessed. Furthermore, the extent to which origin and sex contribute to age prediction variability was investigated, providing insights into the generalizability and robustness of SERS-based ANN models for mosquito age grading.

## Methods

### Mosquitoes

A TH strain of *Ae. aegypti*, derived from field-collected mosquitoes (15°72 N, 101°75 E), has been maintained since 2009 with annual supplementation of first filial generation (F1) eggs to preserve natural genetic heterogeneity. Mosquitoes were reared under controlled conditions, with adults held in an environmental chamber maintained at 28 ± 0.27 °C and 85.09 ± 4.60% relative humidity (RH) with a 14:10 light/dark cycle, as described previously [37]. The physiological age of these mosquitoes, expressed in degree days, was estimated by summing up the hourly difference between the temperature and the development threshold (14.0 °C), divided by 24. Mosquitoes aged 3 days (43 degree days), 10 days (141 degree days), and 18 days (252 degree days) were removed, frozen, and shipped on dry ice from Cornell University (Ithaca, NY) to the University of Massachusetts Amherst (Amherst, MA).

A CA strain of *Ae. aegypti*, derived from field-collected mosquitoes from multiple locations in Los Angeles County (34.06° N, 118.24° W) and Orange County (33.72° N, 117.83° W), has been maintained at MosquitoMate Inc. (Lexington, KY) since 2022. Eggs from this strain were shipped to the Greater Los Angeles County Vector Control District and hatched in shallow plastic trays (Cambro #DB 18263CW148 18 × 26 × 3 inches; Webstaurant, Lititz, PA) filled with 4 L of tap water (approximately 2.5 cm deep) at a density of approximately 6000 larvae per tray. Food capsules (MosquitoMate Inc., Lexington, KY), consisting of 500 mg of liver powder (MB Biomedicals, San Diego, CA) in a cellulose capsule (size 00 vegetarian capsules; Herb Affair, Chicago, IL), were added according to a prescribed schedule. The CA strain was maintained in an environmental chamber with a 14:10 light/dark cycle at 27.0 ± 1.0 °C and 65.0 ± 5.0% RH. Pupae were transferred from larval trays to water cups placed in 30 × 30 × 30 cm cages (BugDorm-1 Insect Rearing Cage, Taiwan) to allow for eclosion. Post-eclosion, adult mosquitoes were provided with 10% sucrose ad libitum. Since hourly temperature was not monitored for the CA strain, physiological age in degree days of these mosquitoes was estimated by summing up the daily difference between the mean set point temperature (27.0 °C) and development threshold (14.0 °C). Adults aged 1 day (13 degree days), 7 days (91 degree days), 14 days (182 degree days), and 21 days (273 degree days) post-emergence were removed, held at −80 °C, and shipped on dry ice from the University of California, Davis (Davis, CA), to the University of Massachusetts Amherst (Amherst, MA).

### Sample preparation

The mosquito water extract was prepared using a modified protocol based on Wang et al. [37] and Gao et al. [38]. Each mosquito was thawed on ice and immersed in 200 µl of ultra-purified water. Homogenization was performed using an ultrasonic disruptor (Branson, Fisher Scientific, Waltham, MA) at a power of 10 W for 30 s on ice. The homogenates were then centrifuged at 10,000×*g* for 5 min at 4 °C, and the supernatant was carefully collected. Next, 20 µl of the supernatant sample was mixed with 20 µl of AgNP colloidal solution (60 nm, citrate coating, NanoComposix) by vortexing. The mixture was incubated for 15 min at room temperature. Finally, 5 µl of the mixture was deposited onto an aluminum foil-covered glass slide and air-dried for SERS analysis.

### Instrumentation

**Fig. 1:**
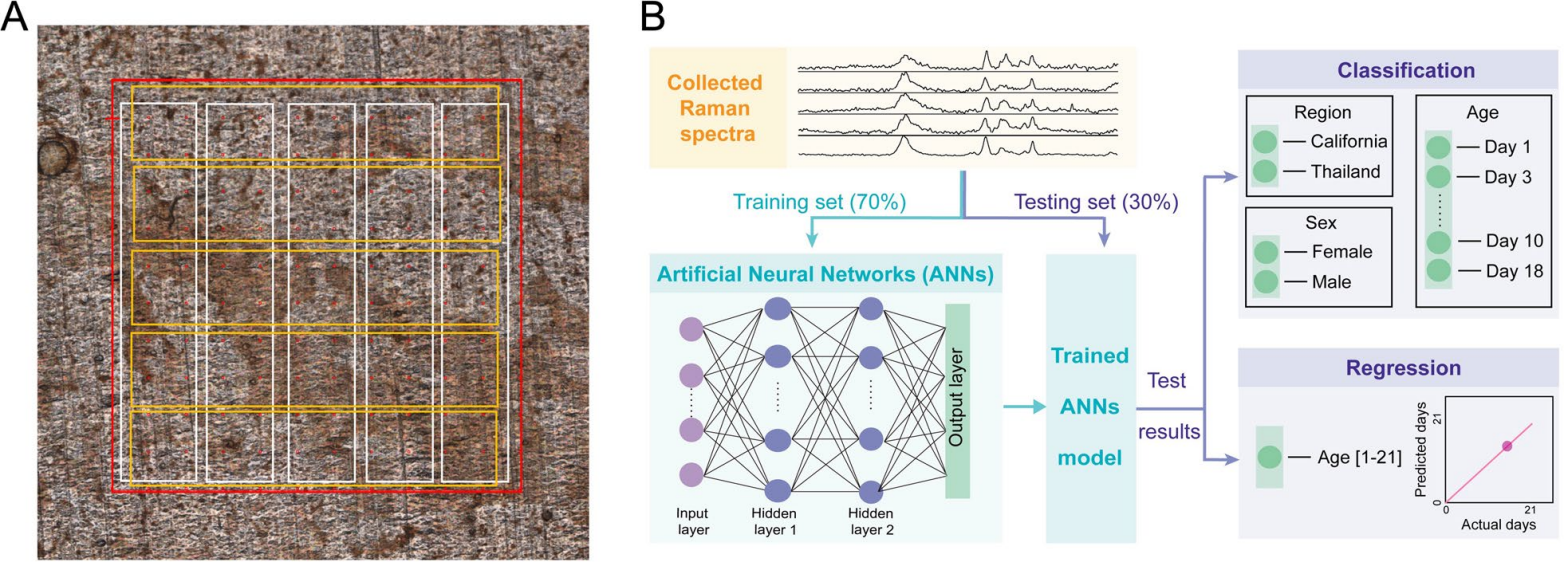
Sample acquisition map and the design and configuration of the ANN model. **A** Sample acquiring area (red square) and 10 sub-areas (white and yellow rectangles) used for data collection. **B** ANN design and configuration for age classification and regression

SERS spectra were captured using a DXRxi Raman microscope (Thermo Fisher Scientific, Madison, WI) equipped with a 780 nm laser and a ×20 objective. For each dried water extract droplet, two 0.9 cm × 0.9 cm areas were chosen for SERS spectra mapping at a laser power of 5 mW and an acquisition time of 1 s. Within each 0.9 cm × 0.9 cm area, 169 spectra were gathered. These spectra were averaged across 10 different rectangular regions (five vertical and five horizontal) (Fig. 1A), yielding 10 averaged spectra per 0.9 cm × 0.9 cm area. Consequently, 20 spectra per mosquito were used for data analysis.

### Data preprocessing and traditional linear chemometric (non-ANN) models

All spectra were normalized using *Z*-score normalization. Norris second derivatives [39] were then applied to reduce noise prior to inputting the data into the models. For classification tasks (i.e., predicting discrete labels such as origin, sex, and categorical age), we used PCA, a traditional linear chemometric model, as a baseline for comparison. Samples were classified based on their proximity to class means using the Mahalanobis distance after PCA projection onto leading principal components. Approximately 70% of the data were used for training, and the remaining 30%, comprising unseen data from different mosquitoes, were reserved for validation. Notably, no training and validation SERS spectra originated from the same mosquito.

For quantitative age-prediction—that is, regression to estimate continuous ages (e.g., 3.2 days), we used PLS. The dataset was split similarly, with 70% used for training and 30% for validation. Model performance was assessed using percentage accuracy (after thresholding the continuous age labels into discrete age bins), the *R*-value, and the RMSE.

### ANNs

Data preprocessing followed a similar approach to that used for non-ANN models. A simple multilayer perceptron (MLP) with two hidden layers, rectified linear unit (ReLU) activation function, and 100 neurons was used for mosquito analysis based on their SERS spectra, as shown in Fig. 1B.

For qualitative analysis (classification), the ANN model was calibrated using a cross-entropy loss function to classify mosquitoes into distinct age categories. For quantitative analysis (Fig. 1B), an ANN regression model was used, with the loss function replaced by mean squared error. The same 70–30 training–validation split was applied. Both models were trained using the full-batch Adam optimizer for 10,000 iterations with an initial learning rate of 0.001. The percentage accuracy, *R*, and RMSE were calculated to evaluate model quality. Additionally, t-distributed stochastic neighbor embedding (t-SNE) and a confusion matrix were used to visualize the results and assess model performance.

### Voting mechanism

Each mosquito in the test set generated 20 individual spectra, with each spectrum independently classified into an age category using the trained model. Given the inherent spectral variability within a single mosquito, a majority voting approach was implemented to enhance classification accuracy. Specifically, if 55% or more (i.e., at least 11 out of 20 spectra) were assigned to a particular age category, the mosquito was classified into that category.

## Results

### Age grading for *Aedes aegypti*, CA-origin

**Fig. 2:**
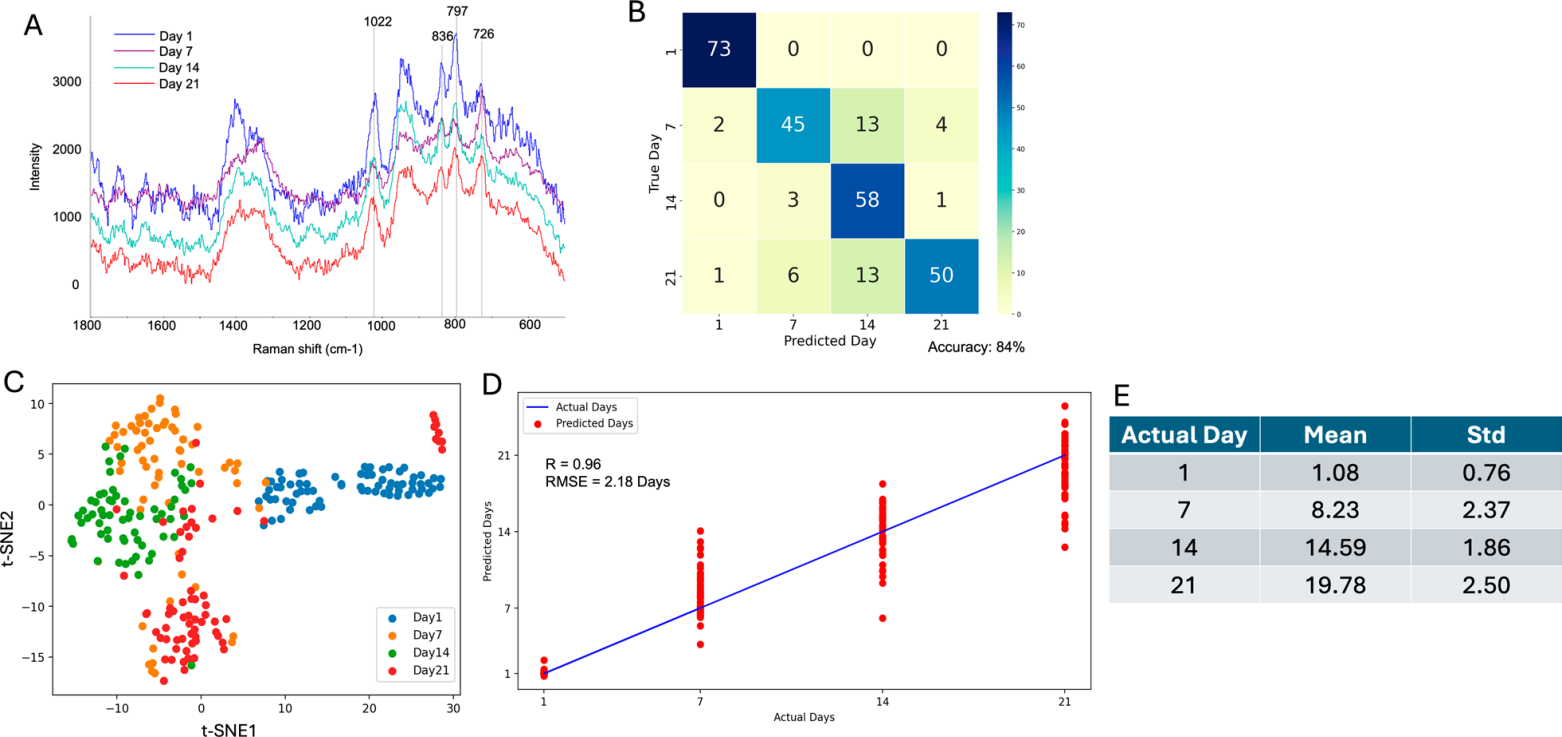
Age classification and regression analysis for mosquitoes collected for the CA-origin. **A** Average SERS spectra of mosquitoes collected on days 1, 7, 14, and 21 at the CA-origin. **B** Confusion matrix showing the accuracy of the model in classifying mosquito samples by collection day. **C** t-SNE plot illustrating the clustering of mosquito samples based on collection day. **D** Regression analysis comparing the actual versus predicted collection days. **E** Predicted mosquito age (mean ± standard deviation)

To investigate the spectral variations associated with mosquito aging, we analyzed the SERS spectra of CA-origin female mosquitoes collected at different ages. The CA-origin (female) was reared at the CA site and collected on day 1 (*n* = 17), day 7 (*n* = 15), day 14 (*n* = 15), and day 21(*n* = 17). In Fig. 2A, the SERS spectra for day 1 are notably distinct from those of the subsequent days, primarily due to the lower intensity observed at the peak of 726 cm^−1^ (adenine). Additionally, the intensities at the 797 cm^−1^ (tryptophan), 836 cm^−1^ (tyrosine), and 1022 cm^−1^ (phenylalanine, C–H in-plane bending) peaks are significantly higher for day 1 than for days 7, 14, and 21. This trend is consistent with our previous observations [37, 38]. Days 7, 14, and 21 display greater similarity in their SERS characteristics, especially when compared to the distinct signature observed for day 1. For instance, the peak intensity at 726 cm^−1^ (adenine) gradually decreased, and peaks at 797 cm^−1^ (tryptophan), 836 cm^−1^ (tyrosine), and 1022 cm^−1^ (phenylalanine, C–H in-plane bending) increased from day 7 to day 14 but decreased on day 21. These patterns suggest that biochemical changes over time result in a convergence in molecular features.

The ANN model demonstrated a strong capability to distinguish between these time points, albeit with some misclassifications. As illustrated in Fig. 2B, the model achieved overall accuracy of 84%, compared to 62% for non-ANN models (Table 1). It accurately classified all 73 spectra from day 1 without misclassifications, and correctly identified 45 out of 64 spectra from day 7, with some overlap in the predictions for days 14 and 21. For day 14, the model correctly identified 58 out of 62 spectra, again with minor misclassifications primarily between days 7 and 21. Day 21 presented the greatest challenge for the model, with 50 out of 70 spectra correctly classified and some predictions incorrectly assigned to day 14. These results suggest that while the model performs well, especially for the earlier days, the spectral similarities between later days (day 14 and day 21) make precise classification more difficult.

**Table 1:** Classification and regression model performance for age grading for mosquitoes of Californian and Thai origins

Origin and sex	Model	Classification		Regression (chronological day)		Regression (degree days)	
		Number of mosquitoes	Accuracy (%)	*R*	RMSE (days)	*R*	RMSE (days)
CA (F)	Non-ANN	64	62	0.84	4.26	0.84	57.30
CA (F)	ANN	64	84	0.96	2.18	0.96	40.00
TH (F)	Non-ANN	30	68	0.88	2.90	0.88	40.46
TH (F)	ANN	30	86	0.95	1.82	0.95	28.47
TH (M)	Non-ANN	30	66	0.82	3.40	0.83	47.30
TH (M)	ANN	30	81	0.94	1.92	0.94	31.00
TH (F & M)	Non-ANN	60	66	0.80	3.67	0.81	51.10
TH (F & M)	ANN	60	83	0.94	2.04	0.94	28.24
CA (F) + TH (F)	Non-ANN	94	50	0.76	4.70	0.76	62.98
CA (F) + TH (F)	ANN	94	80	0.93	2.52	0.91	37.45

The t-SNE analysis (Fig. 2C) further supports these findings. Samples from day 1 (blue dots) form a distinct and well-separated cluster, while samples from days 7 (orange dots), 14 (green dots), and 21 (red dots) form somewhat distinct but overlapping clusters. This overlap likely reflects the gradual molecular changes in the samples over time, making perfect categorical classification more challenging.

To determine the predicted accuracy based on individual mosquitoes rather than individual spectra, a voting mechanism with a 55% threshold was applied (Additional file 1: Table S1). Specifically, each mosquito generated 20 spectra, and each spectrum was independently classified into an age group. If at least 55% (11 out of 20) of the spectra for a mosquito were assigned to the same age, the mosquito was classified into that age group. This method accounts for spectral variability while ensuring robust mosquito-level classification. As shown in Additional file 1: Table S1, all test mosquitoes met this threshold and were correctly assigned to their actual age group, resulting in 100% mosquito-level classification accuracy. This demonstrates the model's reliability in accurately determining mosquito ages across various days. In comparison, when the same voting mechanism was applied to the non-ANN models, the performance was considerably lower (Additional file 1: Table S2). For day 1, all five mosquitoes were correctly predicted, but for day 7, only two out of four were successfully classified. For day 14, no mosquitoes were accurately predicted, while for day 21, four out of five were correctly classified. This resulted in an overall accuracy of 61% for the non-ANN models, further emphasizing the superior performance of the ANN approach.

Due to the difficulty of precise categorical prediction for mosquito age without a voting mechanism, we also evaluated the model's capability to perform continuous age prediction (i.e., allowing outputs such as 4.2 days) through ANN-regression. The scatter plot (Fig. 2D) presents the correlation between actual and predicted ages, with the blue line indicating perfect correlation and the red dots showing model predictions. The regression analysis yielded a high *R*-value of 0.96 and an RMSE of 2.18 days, indicating strong predictive accuracy, especially when compared to the non-ANN models (*R* = 0.84, RMSE = 4.26 days; Table 1). As shown in Fig. 2D, predictions for day 1 closely aligned with actual values, with red dots clustering tightly around the line of perfect correlation. In contrast, predictions for days 7, 14, and 21 displayed greater spread, suggesting decreased precision at higher ages, likely due to overlapping spectral features. To further assess prediction variability, we summarized the mean and standard deviation of predicted ages for each actual day (Fig. 2E). While the predicted means remained close to the true values across all age groups, the standard deviations increased slightly with age. This further supports the observation that spectral similarity among older mosquitoes can make fine-scale age differentiation more difficult.

### Age grading for *Aedes aegypti*, TH-origin

**Fig. 3:**
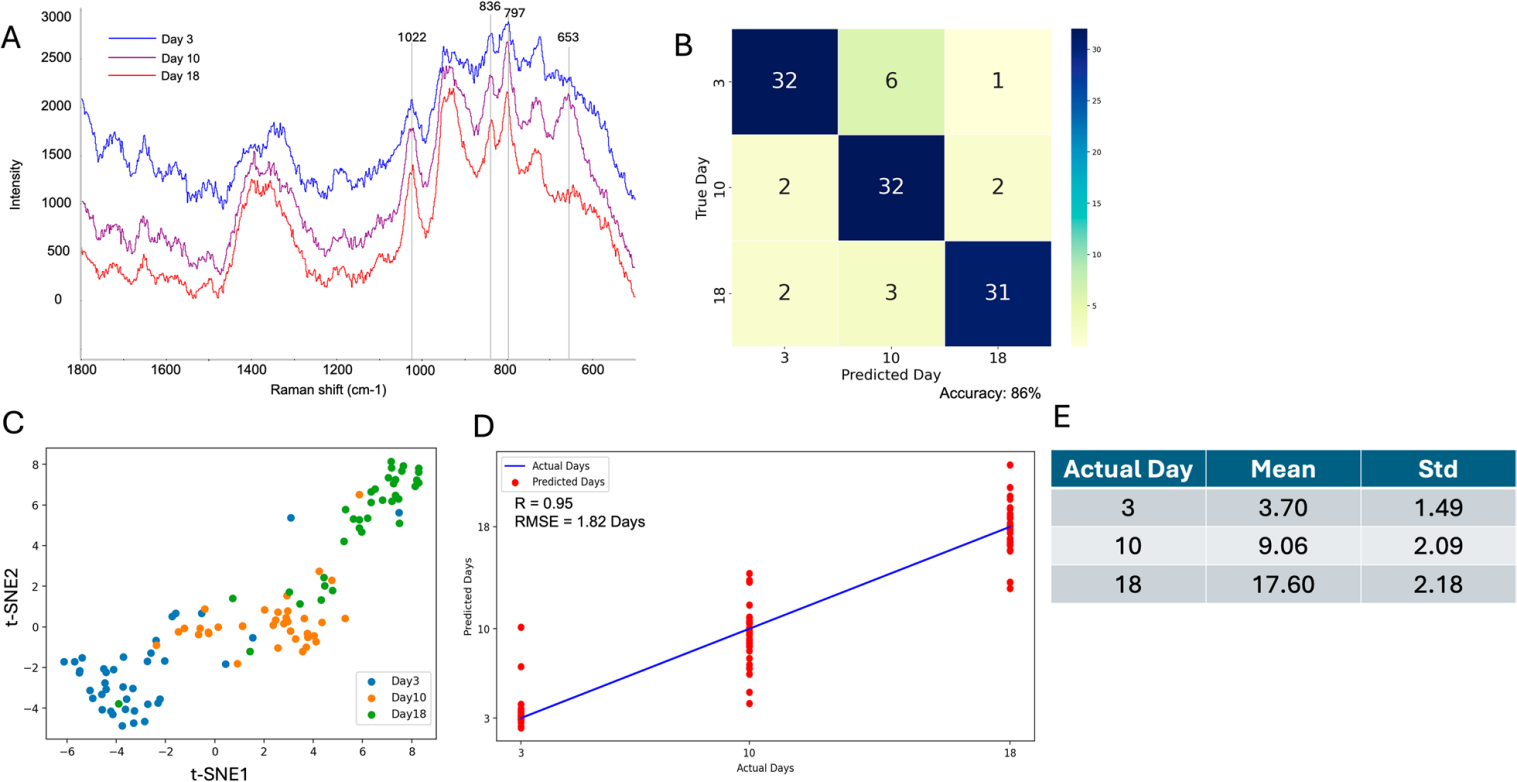
Age classification and regression analysis for mosquitoes collected for the TH-origin. **A** Average SERS spectra of mosquitoes collected on days 3, 10, and 18 at the TH-origin. **B** Confusion matrix showing the accuracy of the model in classifying mosquito samples by collection day. **C** t-SNE plot illustrating the clustering of mosquito samples based on collection day. **D** Regression analysis comparing the actual versus predicted collection days. **E** Predicted mosquito age (mean ± standard deviation)

Similarly, we applied the same model to classify and predict the age of female TH mosquitoes collected on day 3 (*n* = 10), day 10 (*n* = 10), and day 18 (*n* = 10). The SERS spectra (Fig. 3A) revealed distinct biochemical changes over time, with notable differences at the peaks 797 cm^−1^ (tryptophan), 836 cm^−1^ (tyrosine), and 1022 cm^−1^ (phenylalanine, C–H in-plane bending). Particularly noteworthy is the peak at 653 cm^−1^ (C–S stretching), which becomes markedly dominant on day 10. This pattern aligns with our previous findings [38].

As illustrated in Fig. 3B, the model achieved overall accuracy of 86% (Table 1) in classifying TH mosquito samples across the three time points: day 3, day 10, and day 18. The model exhibited consistent classification performance, correctly identifying 32 out of 39 spectra for day 3, 32 out of 36 spectra for day 10, and 31 out of 36 spectra for day 18. When the non-ANN model was used, the overall accuracy decreased to 68%, reflecting its limitations in handling complex spectral data (Table 1). While there were some minor misclassifications, specifically overlaps between days 3 and 10, and between days 10 and 18, the overall results indicate that the model maintained comparable accuracy for each day. This balanced performance suggests that the model is effective in distinguishing between these age groups, even in the presence of similar spectral features.

The t-SNE analysis presented in Fig. 3C further supports these findings, showing distinct clusters for days 3, 10, and 18. There is minimal overlap between the clusters for days 3 and 10, as well as between days 10 and 18. This limited overlap in the t-SNE plot aligns with the high classification accuracy indicated in the confusion matrix, suggesting that the model is effectively distinguishing between the time points based on their unique molecular features.

Using a voting mechanism with a 55% threshold (Additional file 1: Table S1), the model again achieved 100% accuracy for mosquitoes in the test set. In contrast, when the non-ANN model was used, the overall performance decreased to 67% (Additional file 1: Table S3), with three out of three mosquitoes correctly classified for day 3 and day 10, but none successfully predicted for day 18.

Regression analysis (Fig. 3D) showed a strong correlation (*R* = 0.95) and an RMSE of 1.82 days, indicating high predictive accuracy (Table 1). The red dots representing predictions for days 3 and 18 are closely clustered around the line of perfect correlation, indicating good precision for these age groups. In contrast, predictions for day 10 show greater dispersion, suggesting reduced precision likely due to increased spectral variability among intermedia mosquitoes, which can make accurate age differentiation more challenging. This trend is further supported by the summary statistics in Fig. 3E. The predicted means for all age groups remained close to the actual values (3.70, 9.06, and 17.60), but the standard deviation was highest for day 10 (2.09), reflecting more variability in predictions. Compared with the non-ANN model (*R* = 0.88, RMSE = 2.90), the ANN model demonstrated superior capability in handling complex and variable spectral data.

### Impact of sex—age grading for TH *Aedes aegypti,* female and male

We also evaluate the robustness of our model, trained on female mosquitoes, in age grading across sexes. The average SERS spectra for males and females (Additional file 1: Figure S1A) show differences in peak intensities. Females exhibited higher intensities at 1349 cm^−1^ and 726 cm^−1^ (related to adenine), while males showed lower intensities at 1611 cm^−1^ (melanin, C=C stretching) and 631 cm^−1^ (C–C–C bending). Both ANN and t-SNE analysis (Additional file 1: Figure S1B and C) demonstrated a clear spectral separation between male and female samples.

Unsurprisingly, when the model trained on females was applied directly to classify and predict the age of male mosquitoes, as well as for combined male and female samples from the TH site, all performance metrics decreased (Table 1). These results suggest that models trained on mosquitoes of a single sex may not generalize well to a different sex. Therefore, sex-specific data collection is crucial for achieving accurate age predictions across sexes.

### Impact of origin—age grading for all mosquitoes collected from both origins

The average SERS spectra from CA and TH origins (Additional file 1: Figure S2A) clearly reveal distinct molecular fingerprints between these two groups. Specifically, the peaks at 1102 cm^−1^ (C–C/C–N stretching), 892 cm^−1^ (C–H bending), 726 cm^−1^ (adenine), and 631 cm^−1^ (C–C–C bending) are significantly higher in the CA-origin than the TH-origin. These differences suggest variations in the molecular composition and concentration between mosquitoes from the two strains, likely reflecting differences in both geographic origin and laboratory rearing methods. Both ANN models and t-SNE analysis (Additional file 1: Figure S2B and C) further supported these findings.

**Fig. 4:**
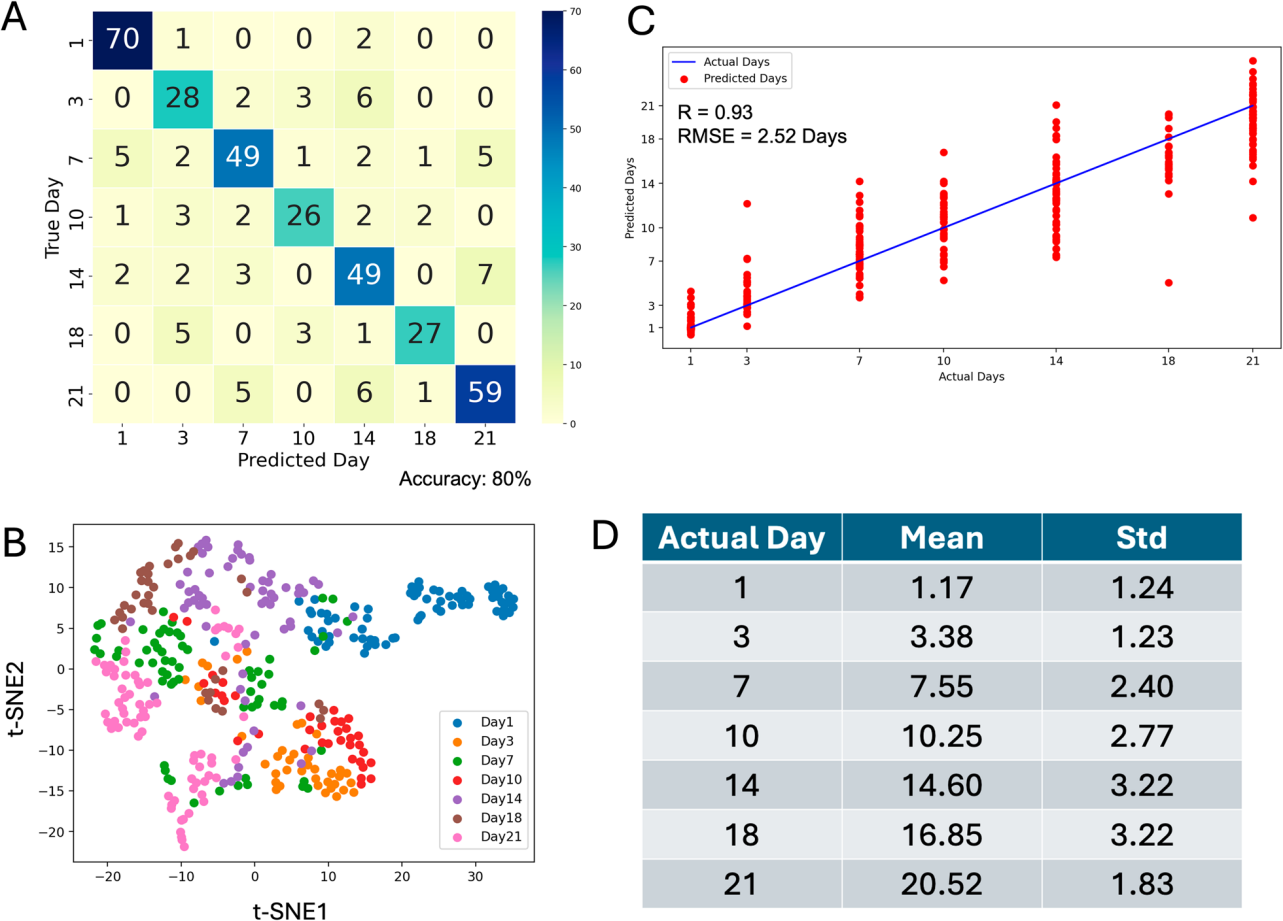
Days classification and regression analysis for mosquitoes collected from both origins. **A** Confusion matrix showing the accuracy of the model in classifying mosquito samples by collection day. **B** t-SNE plot illustrating the clustering of mosquito samples based on collection day. **C** Regression analysis comparing the actual versus predicted collection days. **D** Predicted mosquito age (mean ± standard deviation)

To assess model robustness across different origins, we tested it on all female spectral data from both CA and TH origins. The overall accuracy dropped to 80% (Table 1), lower than the accuracy achieved on individual datasets. Figure 4A shows that the model performed well in classifying day 1 and day 21 samples, correctly identifying 70 out of 73 and 59 out of 71 samples, respectively. However, it struggled with the intermediate days, particularly day 10 and day 18, where spectral similarities led to more misclassifications. The t-SNE plot (Fig. 4B) confirms this, showing more overlap between days 7, 10, 14, and 18 in the combined dataset.

Figure 4C shows the regression analysis, where the model achieved a strong correlation (*R*) of 0.93 and an RMSE of 2.52 days (Table 1). While the predictions for days 1 and 21 are closely aligned with the actual mosquito ages, there is greater variability in the predictions for the intermediate days, reflecting the same challenge observed in the classification task. This trend is further supported by the summary in Fig. 4D, where the standard deviations for intermediate ages (days 7 to 18) are higher—particularly for days 10, 14, and 18—indicating increased spectral variability that complicates precise age prediction in these ranges.

To reduce variations caused by differences in rearing locations, we tested the use of degree days instead of chronological days. While degree days improved accuracy for field-aged mosquitoes in our previous study [38], they had a limited impact on model performance in the current dataset. Using degree days, the *R*-value decreased slightly to 0.91, and the RMSE was 37.45 degree days, approximately equivalent to 2.69 chronological days (Table 1). This indicates that the impact of differing origins cannot be fully mitigated by relying solely on degree days in this case.

Despite the decreased accuracy on individual SERS spectra, applying the same voting mechanism (as previously described) to each individual mosquito, with a threshold set to 55% (see Additional file 1: Table S1), again resulted in 100% accurate classification of mosquitoes in the test set. This demonstrates that the voting strategy effectively mitigates the impact of origin-related variability. In contrast, when the non-ANN model was applied, the overall performance decreased significantly to 37% (Additional file 1: Table S4). The robustness of our model to other domain shifts (such as diverse strains and rearing environments) will be further investigated in future work.

## Discussion

This study highlights the effectiveness of combining SERS with ANN for mosquito age grading. SERS was able to capture age-related biochemical changes in spectral patterns, leading to accurate classification and regression analysis of mosquito ages using ANN. Compared with non-ANN models, the ANN model demonstrated superior predictive power. Non-ANN models relied on linear separability, which limited their ability to distinguish overlapping spectral patterns, leading to reduced accuracy in classification. Similarly, regression-based approaches provided reasonable continuous age predictions but struggled with nonlinear spectral variations, affecting overall performance. In contrast, the ANN model leveraged nonlinear activation functions and deep feature extraction, allowing it to learn complex spectral relationships and achieve significantly higher classification accuracy and regression performance, as reflected in higher *R*-values and lower RMSE.

Consistent with our previous findings (2023) [38], the model effectively differentiated mosquito age groups when trained and tested on the same origin, either CA or TH mosquitoes. For CA mosquitoes, early developmental stages exhibited distinct spectral patterns, while older mosquitoes showed increased molecular similarities, resulting in greater spectral overlap and more challenging classification. In contrast, for TH mosquitoes, the model maintained consistent performance across all age groups. Despite these differences, the ANN model demonstrated strong overall accuracy, underscoring its effectiveness in age grading of mosquitoes within a single origin.

Building on prior work, this study further examines two key aspects: the influence of geographic origin and sex on model accuracy and the implementation of a voting mechanism to enhance age-grading reliability. First, environmental conditions, rearing methods, and genetic background significantly contribute to spectral variability. While normalization techniques such as degree days improved ANN-based age grading across different origins and sexes, they did not fully mitigate these variations, reducing model accuracy. These findings highlight the complexity of factors affecting ANN performance and suggest that future studies explore advanced ML techniques, such as domain adaptation, to further improve model robustness for mosquito age grading across different origins. Second, a voting mechanism was introduced to improve classification reliability by aggregating multiple spectra per mosquito. This approach significantly reduced classification errors, enhancing practical applicability in real-world scenarios where uncertainty and spectral variability impact predictions.

Recent studies have also explored other advanced techniques for mosquito age prediction. For example, matrix-assisted laser desorption/ionization time-of-flight mass spectrometry (MALDI-TOF MS) has been used in combination with deep learning or ANNs. Mohammad et al. [40] used deep learning regression to estimate the age of field-collected *Anopheles arabiensis*, and Nabet et al. [41] applied neural networks to classify *Anopheles stephensi* into discrete age categories. While both studies showed promising accuracy, they require high-end instrumentation and complex sample preparation, which limit their practical use in field settings. Similarly, Mgaya et al. [42] demonstrated that MIR spectroscopy combined with ML could classify *An. arabiensis* into two distinct age groups (5 and 17 days) with high accuracy. However, this approach was restricted to binary classification and lacked the ability to resolve fine-grained age differences across a broader range.

In contrast, the success of our SERS-ANN models suggests a rapid, accurate, and cost-efficient alternative to existing mosquito age-grading methods. Though the bench-top Raman microscope has a high initial cost, the consumables have minimal cost—requiring only 20 µl of AgNP colloidal solution per sample at $0.054. Future experiments will explore a low-cost portable Raman spectrometer. Integrated with ML, SERS enables rapid and precise age differentiation, offering a competitive solution for mosquito age prediction.

However, this study has some limitations. It was conducted under controlled laboratory conditions using mosquitoes from two specific geographic origins. The model’s generalizability to field-collected mosquitoes or populations with different genetic and environmental backgrounds remains to be validated. Additionally, while multiple spectra were analyzed per mosquito, the limited number of individuals per group may not fully capture biological variability. Finally, the current method uses a bench-top Raman microscope; its field utility may be limited until portable Raman systems are evaluated and optimized for this application.

## Conclusions

This study demonstrates the potential of integrating SERS with ANN for mosquito age grading, providing a high level of accuracy in predicting mosquito ages. The model effectively captured spectral variations associated with mosquito aging and outperformed traditional non-ANN approaches. However, several factors, including mosquito origin, environmental conditions, and biochemical differences, influenced classification performance, highlighting the need for further refinements to improve generalizability.

Despite its promise, this approach faces challenges in field applications, including the necessity for standardized calibration across different mosquito strains and the feasibility of real-time deployment. Future research should focus on expanding datasets to include diverse mosquito populations, refining normalization techniques to minimize origin-based variability, and integrating portable Raman spectrometers for field implementation. While additional development and validation are required, this method has the potential to enhance mosquito surveillance and contribute to more effective vector control strategies, ultimately supporting efforts to mitigate mosquito-borne disease transmission.

## Supplementary Information

Additional file 1: Figure S1. Sex classification of mosquitoes. Figure S2. Origin classification of mosquitoes. Table S1. Predicted age distribution for CA and TH strain mosquito test samples using ANN model. Table S2. Predicted age distribution for CA-origin mosquito test samples using non-ANN model. Table S3. Predicted age distribution for TH-origin mosquito test samples using non-ANN model. Table S4. Predicted age distribution for CA- and TH-origin mosquito test samples using non-ANN model

## Data Availability

The dataset generated and source code used in this study have been deposited at https://github.com/gaozili1989 and are publicly available as of the date of publication.

## Abbreviations

SERS: Surface-enhanced Raman spectroscopy
ANN: Artificial neural network
CA: California
ML: Machine learning
TH: Thailand
RMSE: Root mean square error
ReLU: Rectified linear unit
t-SNE: t-Distributed stochastic neighbor embedding
*R*: Correlation coefficient

## Declarations

### Availability of data and materials

The dataset generated and source code used in this study have been deposited at https://github.com/gaozili1989 and are publicly available as of the date of publication.
