# Supplementary material for: Determining mosquito age using surface-enhanced Raman spectroscopy and artificial neural networks: insights into the influence of origin and sex
Source: Parasit Vectors. 2025 Jun 10;18:218. doi: 10.1186/s13071-025-06831-x (PMC12150527; doi:10.1186/s13071-025-06831-x)
Supplement: Supplementary file 1 — Additional file 1: Figure S1. Sex classification of mosquitoes. Figure S2. Origin classification of mosquitoes. Table S1. Predicted age distribution for CA and TH strain mosquito test samples using ANN model. Table S2. Predicted age distribution for CA-origin mosquito test samples using non-ANN model. Table S3. Predicted age distribution for TH-origin mosquito test samples using non-ANN model. Table S4. Predicted age distribution for CA- and TH-origin mosquito test samples using non-ANN model [file 13071_2025_6831_MOESM1_ESM.docx]

**Determining Mosquito Age Using Surface-Enhanced Raman Spectroscopy and Artificial Neural Networks: Insights into the Influence of Origin and Sex**

Zili Gao, Yuzhen Zhang, Laura C. Harrington, Courtney C. Murdock, Elisabeth Martin, Dalton Manbeck-Mosig, Steve Vetrone, Nicolas Tremblay, Christopher M. Barker, John M. Clark, Lili He, Wei Zhu


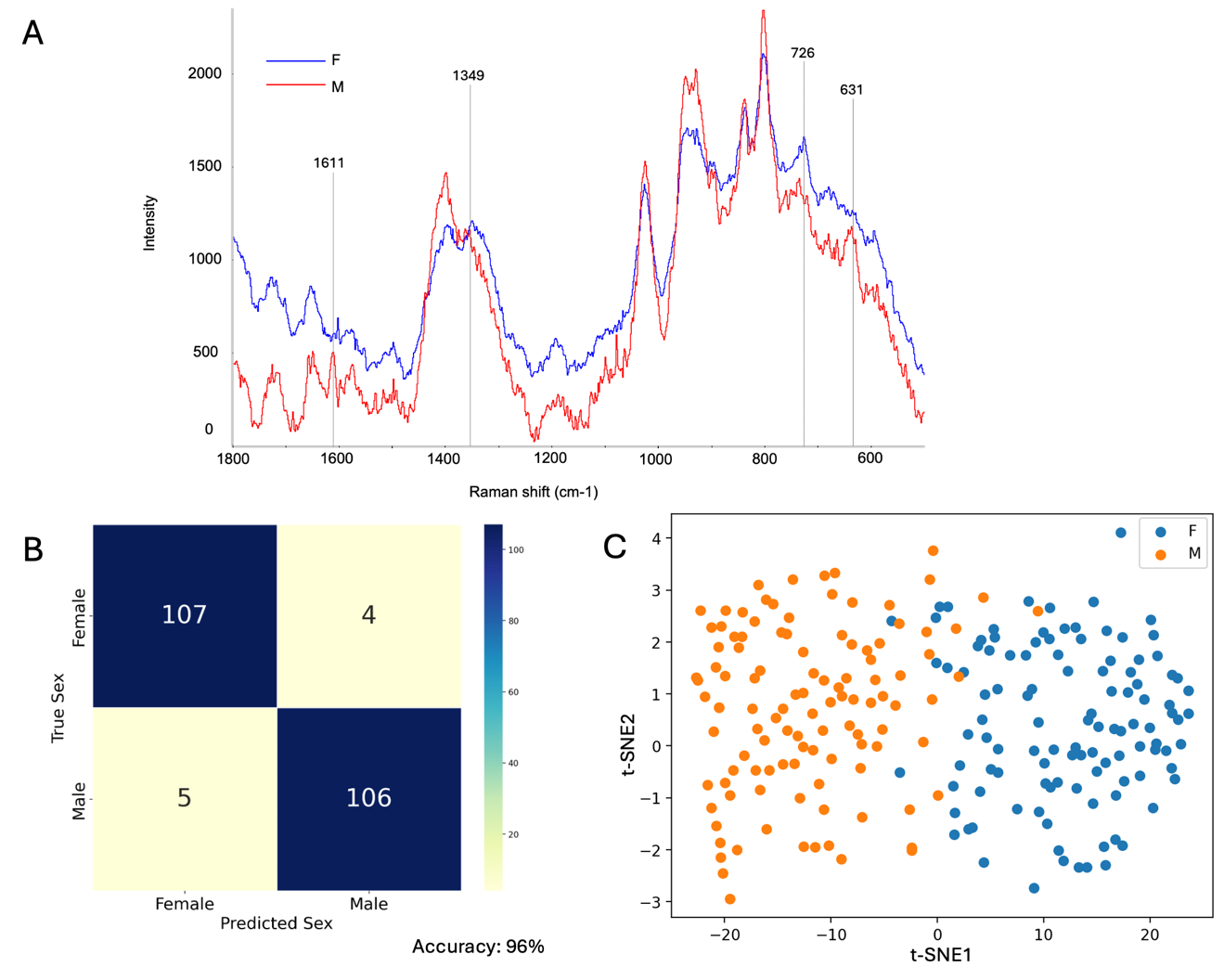


**Figure S1.** Sex Classification of Mosquitoes. **A** Averaged SERS spectra of male and female mosquitoes from TH-origin. **B** Confusion matrix showing the accuracy of the model in sex classification. **C** t-SNE plot illustrating the clustering of mosquito samples based on sex.


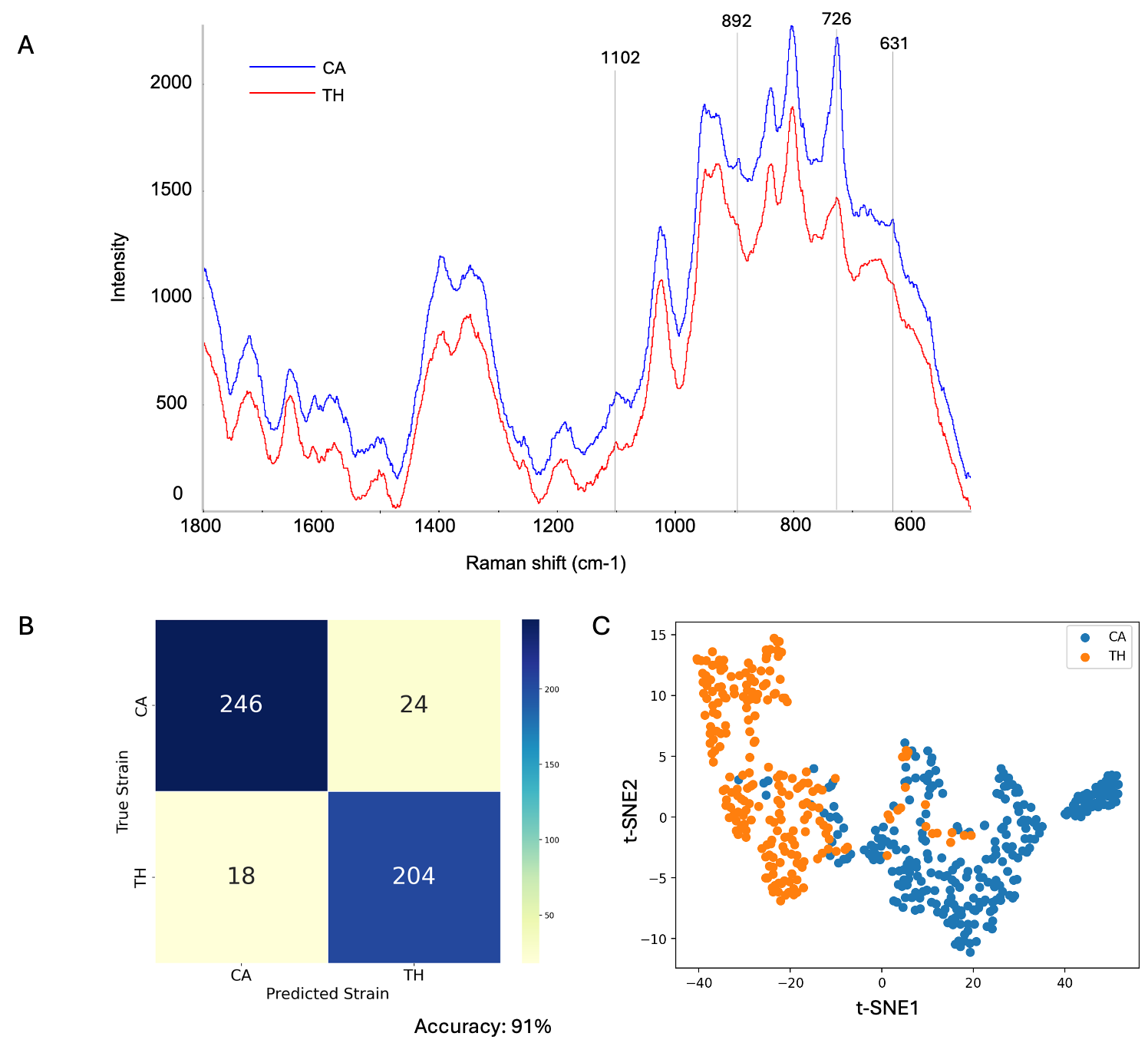


**Figure S2.** Origin Classification of Mosquitoes. **A** Averaged SERS spectra of mosquitoes from CA and TH origins. **B** Confusion matrix showing the accuracy of the model in origin classification. **C** t-SNE plot illustrating the clustering of mosquito samples based on origin.

**Table S1.** Predicted Age Distribution for CA and TH Strain Mosquito Test Samples Using ANN Model

|  | **Predicted** | **Day 1** | **Day 3** | **Day 7** | **Day 10** | **Day 14** | **Day 18** | **Day 21** |
| --- | --- | --- | --- | --- | --- | --- | --- | --- |
| **True** | Mosquitoes Test Set |  |  |  |  |  |  |  |
| **Day 1** | 1 | **89.0%** | 11.0% | 0.0% | 0.0% | 0.0% | 0.0% | 0.0% |
|  | 2 | **100.0%** | 0.0% | 0.0% | 0.0% | 0.0% | 0.0% | 0.0% |
|  | 3 | **100.0%** | 0.0% | 0.0% | 0.0% | 0.0% | 0.0% | 0.0% |
|  | 4 | **94.0%** | 6.0% | 0.0% | 0.0% | 0.0% | 0.0% | 0.0% |
|  | 5 | **100.0%** | 0.0% | 0.0% | 0.0% | 0.0% | 0.0% | 0.0% |
| **Day3** | 1 | 0.0% | **82.0%** | 0.0% | 6.0% | 6.0% | 6.0% | 0.0% |
|  | 2 | 0.0% | **94.0%** | 0.0% | 0.0% | 6.0% | 0.0% | 0.0% |
|  | 3 | 0.0% | **100.0%** | 0.0% | 0.0% | 0.0% | 0.0% | 0.0% |
| **Day 7** | 1 | 0.0% | 7.0% | **71.0%** | 0.0% | 0.0% | 14.0% | 7.0% |
|  | 2 | 11.0% | 0.0% | **61.0%** | 6.0% | 11.0% | 0.0% | 11.0% |
|  | 3 | 0.0% | 0.0% | **94.0%** | 0.0% | 0.0% | 0.0% | 6.0% |
|  | 4 | 0.0% | 0.0% | **60.0%** | 0.0% | 20.0% | 0.0% | 20.0% |
| **Day 10** | 1 | 0.0% | 0.0% | 0.0% | **100.0%** | 0.0% | 0.0% | 0.0% |
|  | 2 | 6.0% | 0.0% | 0.0% | **55.0%** | 5.0% | 5.0% | 29.0% |
|  | 3 | 0.0% | 13.0% | 7.0% | **73.0%** | 0.0% | 0.0% | 7.0% |
| **Day 14** | 1 | 0.0% | 0.0% | 0.0% | 0.0% | **67.0%** | 0.0% | 33.0% |
|  | 2 | 11.0% | 6.0% | 0.0% | 0.0% | **61.0%** | 0.0% | 22.0% |
|  | 3 | 0.0% | 0.0% | 0.0% | 0.0% | **100.0%** | 0.0% | 0.0% |
|  | 4 | 6.0% | 6.0% | 6.0% | 0.0% | **61.0%** | 6.0% | 15.0% |
| **Day 18** | 1 | 0.0% | 0.0% | 15.0% | 30.0% | 0.0% | **55.0%** | 0.0% |
|  | 2 | 0.0% | 6.0% | 0.0% | 0.0% | 0.0% | **94.0%** | 0.0% |
|  | 3 | 0.0% | 0.0% | 0.0% | 17.0% | 0.0% | **83.0%** | 0.0% |
| **Day 21** | 1 | 0.0% | 0.0% | 0.0% | 8.0% | 0.0% | 0.0% | **92.0%** |
|  | 2 | 0.0% | 0.0% | 6.0% | 0.0% | 0.0% | 0.0% | **94.0%** |
|  | 3 | 0.0% | 0.0% | 28.0% | 0.0% | 0.0% | 0.0% | **72.0%** |
|  | 4 | 0.0% | 0.0% | 6.0% | 0.0% | 16.0% | 0.0% | **78.0%** |
|  | 5 | 0.0% | 0.0% | 0.0% | 0.0% | 40.0% | 0.0% | **60.0%** |

**Table S2.** Predicted Age Distribution for CA-origin Mosquito Test Samples Using Non-ANN Model

|  | **Predicted** | **Day 1** | **Day 7** | **Day 14** | **Day21** |
| --- | --- | --- | --- | --- | --- |
| **True** | Mosquitoes Test Set |  |  |  |  |
| **Day 1** | 1 | **65.0%** | 0.0% | 0.0% | 35.0% |
|  | 2 | **88.0%** | 0.0% | 0.0% | 12.0% |
|  | 3 | **100.0%** | 0.0% | 0.0% | 0.0% |
|  | 4 | **94.0%** | 0.0% | 0.0% | 6.0% |
|  | 5 | **100.0%** | 0.0% | 0.0% | 0.0% |
| **Day 7** | 1 | 0.0% | **75.0%** | 0.0% | 25.0% |
|  | 2 | 18.0% | **29.0%** | 12.0% | 41.0% |
|  | 3 | 0.0% | **24.0%** | 0.0% | 76.0% |
|  | 4 | 6.0% | **82.0%** | 0.0% | 12.0% |
| **Day 14** | 1 | 6.0% | 6.0% | **31.0%** | 57.0% |
|  | 2 | 12.0% | 24.0% | **12.0%** | 52.0% |
|  | 3 | 0.0% | 35.0% | **12.0%** | 53.0% |
|  | 4 | 0.0% | 58.0% | **17.0%** | 25.0% |
| **Day 21** | 1 | 0.0% | 40.0% | 20.0% | **40.0%** |
|  | 2 | 0.0% | 6.0% | 0.0% | **94.0%** |
|  | 3 | 0.0% | 0.0% | 0.0% | **100.0%** |
|  | 4 | 0.0% | 24.0% | 0.0% | **76.0%** |
|  | 5 | 7.0% | 14.0% | 7.0% | **72.0%** |

**Table S3.** Predicted Age Distribution for TH-origin Mosquito Test Samples Using Non-ANN Model

|  | **Predicted** | **Day 3** | **Day 10** | **Day 18** |
| --- | --- | --- | --- | --- |
| **True** | Mosquitoes Test Set |  |  |  |
| **Day3** | 1 | **100.0%** | 0.0% | 0.0% |
|  | 2 | **82.0%** | 18.0% | 0.0% |
|  | 3 | **80.0%** | 20.0% | 0.0% |
| **Day 10** | 1 | 25.0% | **75.0%** | 0.0% |
|  | 2 | 18.0% | **76.0%** | 6.0% |
|  | 3 | 14.0% | **86.0%** | 0.0% |
| **Day 18** | 1 | 60.0% | 10.0% | **30.0%** |
|  | 2 | 59.0% | 0.0% | **41.0%** |
|  | 3 | 67.0% | 0.0% | **33.0%** |

**Table S4.** Predicted Age Distribution for CA and TH Origin Mosquito Test Samples Using Non-ANN Model

|  | **Predicted** | **Day 1** | **Day 3** | **Day 7** | **Day 10** | **Day 14** | **Day 18** | **Day 21** |
| --- | --- | --- | --- | --- | --- | --- | --- | --- |
| **True** | Mosquitoes Test Set |  |  |  |  |  |  |  |
| **Day 1** | 1 | **88.0%** | 0.0% | 12.0% | 0.0% | 0.0% | 0.0% | 0.0% |
|  | 2 | **53.0%** | 0.0% | 0.0% | 0.0% | 0.0% | 0.0% | 47.0% |
|  | 3 | **88.0%** | 0.0% | 0.0% | 0.0% | 6.0% | 0.0% | 6.0% |
|  | 4 | **80.0%** | 0.0% | 0.0% | 0.0% | 0.0% | 0.0% | 20.0% |
|  | 5 | **70.0%** | 20.0% | 0.0% | 0.0% | 10.0% | 0.0% | 0.0% |
| **Day3** | 1 | 0.0% | **100.0%** | 0.0% | 0.0% | 0.0% | 0.0% | 0.0% |
|  | 2 | 6.0% | **53.0%** | 6.0% | 0.0% | 6.0% | 0.0% | 29.0% |
|  | 3 | 41.0% | **47.0%** | 6.0% | 0.0% | 6.0% | 0.0% | 0.0% |
| **Day 7** | 1 | 0.0% | 0.0% | **14.0%** | 0.0% | 0.0% | 0.0% | 86.0% |
|  | 2 | 0.0% | 0.0% | **23.0%** | 0.0% | 12.0% | 0.0% | 65.0% |
|  | 3 | 0.0% | 0.0% | **18.0%** | 0.0% | 0.0% | 0.0% | 82.0% |
|  | 4 | 0.0% | 0.0% | **100.0%** | 0.0% | 0.0% | 0.0% | 0.0% |
| **Day 10** | 1 | 0.0% | 0.0% | 12.0% | **0.0%** | 0.0% | 0.0% | 88.0% |
|  | 2 | 6.0% | 35.0% | 0.0% | **47.0%** | 0.0% | 0.0% | 12.0% |
|  | 3 | 0.0% | 37.0% | 9.0% | **18.0%** | 9.0% | 0.0% | 27.0% |
| **Day 14** | 1 | 0.0% | 0.0% | 17.0% | 0.0% | **50.0%** | 0.0% | 33.0% |
|  | 2 | 0.0% | 0.0% | 24.0% | 0.0% | **24.0%** | 0.0% | 52.0% |
|  | 3 | 0.0% | 0.0% | 6.0% | 0.0% | **47.0%** | 0.0% | 47.0% |
|  | 4 | 6.0% | 0.0% | 24.0% | 0.0% | **24.0%** | 0.0% | 46.0% |
| **Day 18** | 1 | 18.0% | 35.0% | 24.0% | 0.0% | 0.0% | **6.0%** | 17.0% |
|  | 2 | 0.0% | 6.0% | 47.0% | 0.0% | 6.0% | **0.0%** | 41.0% |
|  | 3 | 0.0% | 0.0% | 0.0% | 0.0% | 50.0% | **50.0%** | 0.0% |
| **Day 21** | 1 | 7.0% | 0.0% | 20.0% | 0.0% | 0.0% | 0.0% | **73.0%** |
|  | 2 | 29.0% | 0.0% | 24.0% | 0.0% | 0.0% | 0.0% | **47.0%** |
|  | 3 | 6.0% | 0.0% | 0.0% | 0.0% | 0.0% | 0.0% | **94.0%** |
|  | 4 | 0.0% | 0.0% | 41.0% | 0.0% | 0.0% | 0.0% | **59.0%** |
|  | 5 | 0.0% | 0.0% | 0.0% | 0.0% | 43.0% | 0.0% | **57.0%** |
